# Structural conversion of three copper(ii) complexes with snapshot observations based on the different crystal colours and morphology†

**DOI:** 10.1039/d0ra07231a

**Published:** 2020-11-26

**Authors:** Hao Su, Zhongkui Li, Junrui Tan, Hongwei Ma, Li Yan, Hui Li

**Affiliations:** Key Laboratory of Cluster Science of Ministry of Education, School of Chemistry and Chemical Engineering, Beijing Institute of Technology Beijing 100081 P. R. China lihui@bit.edu.cn; Analytical and Testing Center, Beijing Institute of Technology Beijing 102488 China

## Abstract

Three novel Cu(ii) complexes [Cu_2_(L)_2_(MeOH)_2_] (1), [Cu_2_(L)_2_(H_2_O)_2_] (2) and [CuL(H_2_O)] (3) (L = (*E*)-2-((2-hydroxy-4-methoxybenzylidene)amino)acetic acid) have been obtained in different time scales of reaction processing. Complexes 1 and 2 are kinetically controlled products and 3 is a thermodynamically stable product. Single crystal X-ray diffraction analyses revealed that 1 and 2 are binuclear complexes except for different coordination solvents. 3 is a mononuclear complex. Complex 1 is mainly obtained in methanol solution, while 2 and 3 are stable in aqueous solvents. Based on the understanding of crystal structures of the three complexes, reversibly transforming crystal 2 to crystal 1 at room temperature has been realised, which has been confirmed by the change of colours and morphology measured by SEM. The research work is very important for controllable synthesis of coordination complexes.

## Abstract 2

Structural conversion of three novel Cu(ii) complexes [Cu_2_(L)_2_(MeOH)_2_] (1), [Cu_2_(L)_2_(H_2_O)_2_] (2) and [CuL(H_2_O)] (3) (L = (*E*)-2-((2-hydroxy-4-methoxybenzylidene)amino)acetic acid) in different time scales of reaction processing.

## Graphical abstract

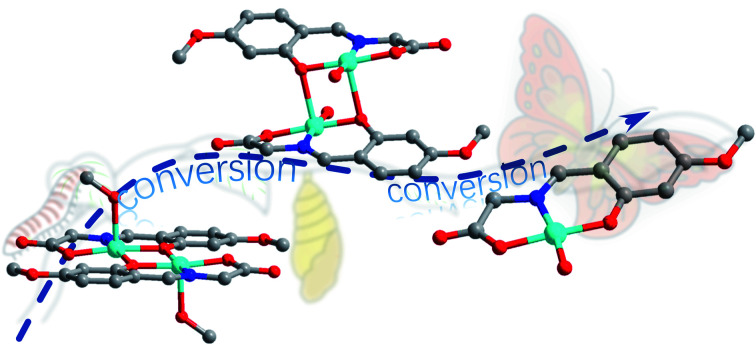

## Introduction

The structural transformation of complexes can lead to different properties, such as colour, morphology, adsorption, chirality, luminescence and magnetism.^1–13^ The potential application value brought by the diversity of complex structures has attracted more and more attention.^14^ The structural transformation is usually characterized by small changes in the number and order of uncoordinated molecules at the molecular level, such as the addition and removal of solvents or guest molecules.^15–17^ For the coordination centre of the unstable kinetic products, because the metal coordination bonds easily break and generate new coordination bonds, deeper structural changes can occur, such as the exchange of ligands or metal ions and the change of coordination number or coordination configuration.^18–21^ Up to mow, it is still a difficult process to predict the synthesis and crystallization of complexes, which is mainly due to the lack of basic understanding of the crystal growth process of complexes and the influence of various experimental parameters on the structure of complexes.^22^ The structural evolution law and stability can be more clearly understood by observing and studying the structural conversion process in the crystallization process.

The self-assembly process of complexes provides not only thermodynamic products but also kinetic products, which is similar to organic reactions.^23^ The trapped local energy of two products is obviously different.^24^ In this process, the kinetic product can be used as the intermediate product of whole reaction. In principle, the structures of stable final products are quite different due to their different activation energies. The formation of kinetic products is rapid and usually occurs at a lower temperature, while the formation of thermodynamic products is slow or at a higher temperature.^25–27^ However, it is difficult to identify or separate the two products in single crystal state because of the difficulty of single crystal growth. Generally, it is difficult to capture or only capture one dynamic product as an intermediate product.^28–31^ Identifying the crystal structure of dynamic product can provide a basis for fully understanding the synthesis process of single crystal. Two continuous intermediate state kinetic products and one final state thermodynamic product were captured for the first time, which provided an excellent basis for exploring the structural evolution of complexes.

**Scheme 1:**
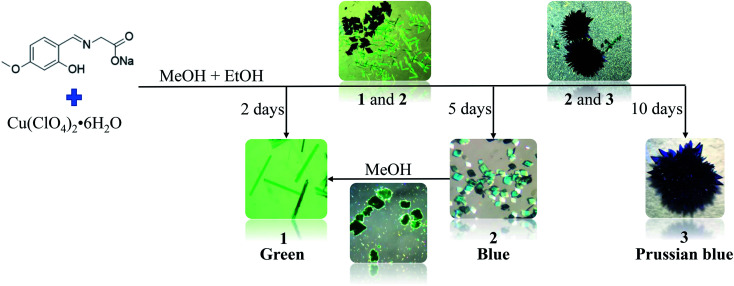
Synthetic procedures and crystal conversion of three complexes 1, 2 and 3.

In our previous work, we have designed the synthesis a novel kind of ligands of 5-substituents of salicylaldehyde modified glycine, which are tridentate ligands. Some coordination complexes of this kind of ligands are three-dimension coordination polymer with crystal structure similar with MOF-74 structure.^32,33^ In this work, as an extension of our study, a new ligand, L = (*E*)-2-((2-hydroxy-4-methoxybenzylidene)amino) acetic acid has been design and synthesized. Its three novel Cu(ii) complexes ([Cu_2_(L)_2_(MeOH)_2_] (1), [Cu_2_(L)_2_(H_2_O)_2_] (2) and [CuL(H_2_O)] (3)) were obtained in the same reaction beaker at room temperature (Scheme 1). The dynamic conversion of complexes 1, 2 and 3 have observed. Importantly, the reversible conversion of complexes 1 and 2 has been realized, which confirm further that 1 and 2 are complexes formed kinetically.

Single crystal X-ray diffraction analyses revealed that 1 and 2 are binuclear complexes except for different coordination solvents. 3 is a mononuclear complex and the coordination solvent of water molecule is the same with 2 so that the molecular structure of 3 is half of 2. With the change of solution content, complex 1 gradually transformed to 2 and finally to 3 with the change of colours in the crystallization process at room temperature, which is a dynamically controlled process with the reaction moving into a more stable direction. Interestingly, 2 and 3 are stable in aqueous solvents, while 2 can be converted to 1 with crystal to crystal in methanol solvents.

## Experimental

### Materials

Copper(ii) perchlorate hexahydrate, 2-hydroxy-4-methoxy-benzaldehyde and glycine were obtained from commercial sources (Aladdin) and used without further purification. All analytical grade organic solvents were purchased from commercial sources (Beijing Chemical) and used as received.

### Physical measurements

^1^H NMR spectra were taken on a 400 MHz Bruker FT-NMR spectrometer and MeOH-d_4_ was used as the solvent. The FT-IR (KBr pellet) was obtained (4000 to 400 cm^−1^ region) by a Nicolet 360 FT-IR spectrometer. Powder X-ray diffraction (PXRD) patterns were carried out on a Bruker SMART APEX charge-coupled device (CCD)-based diffractometer at room temperature (298 K). Elemental analysis (C, H and N) were obtained by an EA3000 elemental analyzer. Absorbance spectra was measured on a Persee TU-1950 spectrophotometer. The scanning electron microscopy (SEM) images were obtained *via* Hitachi JSM-7500F scanning electron microscopy.

### Synthesis of ligand L

The ligand L was synthesized by using glycine, NaOH and 2-hydroxy-4-methoxybenzaldehyde. A stirred mixture of glycine (0.375 g, 5 mmol) and NaOH (0.2 g, 5 mmol) in ethanol (15 mL) was refluxed for 30 min. Then an ethanol solution of 2-hydroxy-4-methoxybenzaldehyde (0.76 g, 5 mmol) was added drop by drop. Next, the mixture was stirred and refluxed for another 5 hours. Yellow precipitate was filtered and washed with ethanol. Yield 86.3%. Anal. calcd for C_10_H_10_NO_4_Na: C, 51.95; H, 4.33; N, 6.06. Found: C, 51.93; H, 4.35; N, 6.02. ^1^H NMR (400 MHz, MeOH-d_4_) *δ* (ppm): 3.79 (s, 3H), 4.16 (s, 2H), 6.15 (s, 1H), 6.17 (d, 1H), 7.09–7.12 (d, 1H), 8.06 (s, 1H). ^1^H NMR and FT-IR spectrum of L are shown in Fig. S1 and S2† respectively.

### Preparation of complex 1, 2 and 3

[Cu_2_(L)_2_(MeOH)_2_] (1) was synthesized *via* a method of room temperature evaporation. A mixture of Cu(ClO_4_)_2_·6H_2_O (0.037 g, 0.1 mmol) and L (0.023 g, 0.1 mmol) in 3 mL methanol and 2 mL ethanol was stirred for 30 min at room temperature and then filtered. The green colour and rod-like single crystals suitable for single-crystal X-ray diffraction analysis were obtained after 2 days by evaporation at room temperature (yield: 33.8.2%, based on Cu). Anal. calcd for C_22_H_26_Cu_2_N_2_O_10_: C, 43.60; H, 4.29; N, 4.62. Found: C, 43.58; H, 4.26; N, 4.64. ATR-FTIR (*v*_max_): 3425m, 2976w, 2854w, 2368w, 2330w, 1609s, 1494m, 1384s, 1275w, 1238w, 1212w, 1141w, 1087w, 1042w, 983w, 933w, 883w, 822w, 792w, 661w, 631w, 601w cm^−1^.

[Cu_2_(L)_2_(H_2_O)_2_] (2) was synthesized by following a same procedure to that of 1 except that the reaction time is different. When the reaction lasted 4 days, the two kinds of crystals of 1 and 2 co-existed, and the product was completely transformed to 2 after 5 days, while blue colour and rhombic single crystals suitable for single-crystal X-ray diffraction analysis were obtained at room temperature (yield: 31.2%, based on Cu). Anal. calcd for C_20_H_22_Cu_2_N_2_O_10_: C, 41.56; H, 3.81; N, 4.85. Found: C, 41.52; H, 3.83; N, 4.86. ATR-FTIR (*v*_max_): 3431 s, 2917w, 2847w, 2364w, 2333w, 1629 s, 1539m, 1458m, 1378 s, 1116w, 1076w, 982w, 933w, 885w, 821w, 792w, 664w, 632w, 603w cm^−1^.

[CuL(H_2_O)] (3) was obtained by following a same procedure to that of 1 and 2 except that the reaction time is different. When the reaction lasted 8 days, the two kinds of crystals of 2 and 3 co-existed, and the product was completely transformed to 3 after 10 days, while Prussian blue colour and blocky single crystals suitable for single-crystal X-ray diffraction analysis were obtained at room temperature (yield: 28.6%, based on Cu). Anal. calcd for C_10_H_11_CuNO_5_: C, 41.56; H, 3.81; N, 4.85. Found: C, 41.58; H, 3.84; N, 4.83. ATR-FTIR (*v*_max_): 3431s, 2921w, 2848w, 2372w, 2332w, 1633s, 1539w, 1458w, 1384s, 1118w, 1074w, 981w, 935w, 887w, 819w, 794w, 667w, 635w, 607w cm^−1^.

### Single crystal X-ray diffraction

Suitable single crystals with dimensions of for compounds 1 (0.10 × 0.15 × 0.25 mm), 2 (0.11 × 0.15 × 0.15 mm) and 3 (0.12 × 0.23 × 0.24 mm) were selected for single-crystal X-ray diffraction analysis respectively. Data collection were obtained on a Bruker AXS CCD area detector with graphite monochromatic molybdenum Kα (*λ* = 0.71073 Å) radiation at room temperature. Unit-cell parameters were determined from automatic centering and refined by the least-squares method. The crystal structure was solved using direct methods and refined by full-matrix least square techniques on *F*^2^*via* the SHELXTL program.^34^ All non-hydrogen atomic positions were located on different Fourier maps and refined anisotropically. Some hydrogen atoms were placed at their geometrically generated positions and some hydrogen atoms were located on different Fourier maps and refined isotropically. Crystallographic data are given in Table 1.

**Table 1:** Crystallographic data for 1, 2 and 3

Compound	1	2	3
Formula	C_22_H_26_Cu_2_N_2_O_10_	C_20_H_22_Cu_2_N_2_O_10_	C_10_H_11_CuNO_5_
*M* (mol^−1^)	605.53	577.47	288.74
*T* (K)	296(2)	296(2)	296(2)
Crystal system	Monoclinic	Monoclinic	Orthorhombic
Space group	*P*2_1_/*c*	*P*2_1_/*c*	*Pbca*
*a* (Å)	9.7692(18)	9.735(2)	7.4851(4)
*b* (Å)	6.5564(12)	8.646(2)	13.4109(7)
*c* (Å)	19.064(4)	13.038(3)	21.5161(11)
*α* (°)	90.00	90.00	90.00
*β* (°)	91.926(7)	96.096(7)	90.00
*γ* (°)	90.00	90.00	90.00
*V* (Å^3^)	1220.4(4)	1091.2(4)	2159.8(2)
*Z*	2	2	8
*ρ* (calculated)(g cm^−3^)	1.648	1.758	1.776
*F* (000)	620	588	1064
*θ* range (°)	2.086–26.998	2.104–25.049	1.893–26.988
GOF on *F*^2^	1.081	1.038	1.062
*R*_int_	0.0549	0.1397	0.0295
*R*_1_ [*I* > 2*σ*(*I*)]^a^	0.0367	0.0778	0.0385
w*R*_2_[*I* > 2*σ*(*I*)]^b^	0.0768	0.1955	0.1115
*R*_1_ (all data)^a^	0.0632	0.1399	0.0471
w*R*_2_ (all data)^b^	0.0841	0.2237	0.1167

a *R*_1_ = ∑||*F*_0_| − |*F*_C_||/Σ|*F*_0_|.

b w*R*_2_ = ∑[*w*(*F*_0_^2^ − *F*_C_^2^)^2^]/Σ[*w*(*F*_0_^2^)^2^]^1/2^.

### Crystal structures

2006301 (1), 2006302 (2) and 2006303 (3) contain the ESI crystallographic data† for this paper.

## Results and discussion

### Syntheses and general characterization

Three compounds 1, 2 and 3 were obtained by the reaction of L with Cu(ClO_4_)_2_·6H_2_O under the same ambient condition in one reaction, while the change of solution content is accompanied by the difference of product formation time (Scheme 1).The ligand is neutralized by sodium hydroxide in the synthesis process. It should be noted that solution content played an important role in the coordination mode of Cu(ii) ions and the growth of compounds 1, 2 and 3. A mixed solvent of methanol and ethanol was used in the synthesis of compounds 1, 2 and 3. The difference between them was that compound 1 was first formed in the synthesis process, and compound 1 gradually transformed into compound 2 with the decrease of solvent and finally to 3 with the change of colours and morphology. The change of solution content induced 2 and 3 are stable in aqueous solvents, while 2 can be converted to 1 with crystal to crystal in methanol solvents. According to the infrared spectrum data and crystallographic analysis results, all carboxyl and hydroxyl groups in 1–3 are found to be deprotonated and coordinated. The coordination modes of ligand L in the three complexes are the same, while Cu(ii) ions have two different coordination modes (Fig. S3†). Cu(ii) is five coordinated in the compounds 1 and 2, and four coordinated in the compound 3, which exert a very important influence on the crystal structure as describe in the following parts.

The powder X-ray diffraction (PXRD) of match up with the simulation data of their single crystals (Fig. S4†), which are synthesized in a facile manner by slow evaporation at room temperature. Thermogravimetric analysis (TGA) showed the decomposition temperatures of 1, 2 and 3 to be 242 °C, 261 °C and 243 °C respectively in a nitrogen atmosphere, which exhibited the weight loss process (Fig. S5†). The losses of 1, 2 and 3 in the first stage were 10.39%, 6.59% and 6.46% respectively, which was attributed to the preferential loss of solvent molecules and was consistent with the theoretical value.

### Crystal structural of [Cu_2_(L)_2_(MeOH)_2_] (1)

**Fig. 1:**
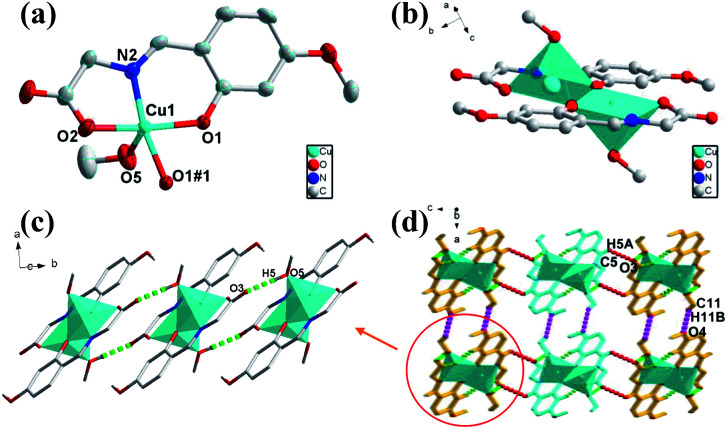
(a) Views of 1 ORTEP representation showing the local coordination environment around the Cu(ii) center with 30% thermal ellipsoid probability. Hydrogen atoms have been omitted for clarity. (b) The binuclear structure of 1. (c) The 1D chain structure with H-bonding from *c* axis. (d) Viewing of the 3D structure with H-bonding from *b* axis.

The green crystal 1 crystallizes in monoclinic space group of *P*2_1_/*c* with an asymmetric unit that contains one Cu(ii) ion, one deprotonated ligand and one methanol molecule (O5) (Fig. 1a). The five-coordinated Cu1 atom is surrounded by O atoms and N atom with distorted square-pyramidal coordination geometry, consisting of three oxygen atoms (Cu(1)–O(1), Cu(1)–O(1#1), Cu(1)–O(2) are 1.966, 1.998 and 1.958 Å respectively) and one nitrogen atom from one ligand (Cu(1)–N(2) is 1.929 Å) in the equatorial position and one oxygen atom from the methanol molecule (Cu–O bond length of Cu(1)–O(5) is 2.252 Å) of occupying the apical position. The binuclear structure formed by two ligands and two Cu atoms (Cu⋯Cu: 3.061 Å) share the phenoxy oxygen atoms, and two ligand molecules of the binuclear structure are in the same plane (Fig. 1b). Only one oxygen atom of the carboxylic acid group participates in the coordination in this compound, while the other oxygen atom in the ligand does not, which causes the entire structure to fail to form a coordination network. In addition, as a basic repeating unit, the adjacent binuclear structures are further bridged by H-bonds (O5–H5⋯O3: 1.829 Å, 2.648 Å, 178.48°) to result an infinite 1D chain structure (Fig. 1c). The adjacent 1D chains through two kinds of weak H-bonds (C11–H11B⋯O4: 2.644 Å, 3.488 Å, 146.94°; C5–H5A⋯O3: 2.582 Å, 3.246 Å, 128.78°) and van der Waals interactions are further assembled into a 3D supramolecular structure in the form of ABAB (Fig. 1d).

### Crystal structural of [Cu_2_(L)_2_(H_2_O)_2_] (2)

**Fig. 2:**
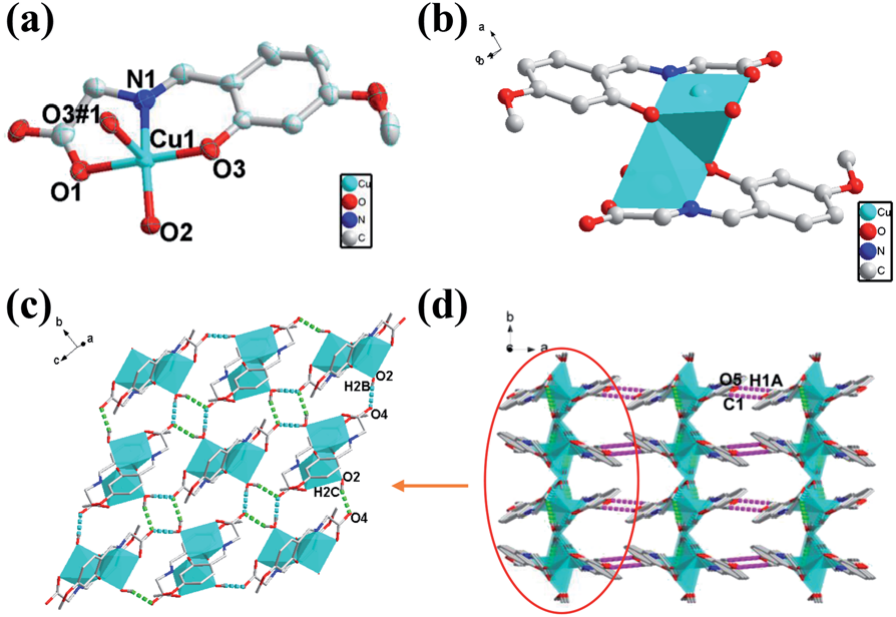
(a) Views of 2 ORTEP representation showing the local coordination environment around the Cu(ii) center with 30% thermal ellipsoid probability. Hydrogen atoms have been omitted for clarity. (b) The binuclear structure of 2. (c) The 2D layer polyhedron structure picture viewed down from *c* axis. (d) Viewing of the 3D structure with H-bonding from *c* axis.

Single crystal X-ray crystallographic analysis reveals that the crystal structure of 2 crystallizes in the monoclinic space group of *P*2_1_/*c* with an asymmetric unit that contains one Cu(ii) ions, one deprotonated ligand and one water molecule. As shown in Fig. 2a, Cu(ii) center of 2 is in a NO4 distorted square-pyramidal coordination geometry, containing two oxygen atoms from one ligand and water molecule (Cu–O bond lengths of Cu(1)–O(1), Cu(1)–O(3), Cu(1)–O(2) are 1.962, 1.907 and 1.959 Å respectively) and one imino N atom from one ligand (Cu–N bond length of Cu(1)–N(1) is 1.919 Å) in the equatorial positions, as well as an oxygen atom from the other ligand in the axial position (Cu–O bond lengths of Cu(1)–O(3#1) is 2.433 Å). The Cu(ii) center of 2 is completely different from that of 1 mainly in the coordination position of solvent molecules. The binuclear structure of 2 formed by two ligands and two Cu atoms (Cu⋯Cu: 3.088 Å) share the phenoxy oxygen atoms, which is in the different plane and different with that of 1 (Fig. 2b). The Cu–O bond for solvent molecule coordination of 2 is shorter than that of 1 because of the coordinate position of the solvent molecule. Unlike 1, the adjacent binuclear structures of 2 as a basic repeating unit are constructed through two kinds of H-bonds (O2–H2B⋯O4: 1.842 Å, 2.683 Å, 169.51°; O2–H2C⋯O4: 1.897 Å, 2.697 Å, 156.22°) to result an infinite 2D supramolecular layer framework (Fig. 2c). The adjacent 2D layers are further constructed into a 3D supramolecular structure with weak H-bonds (C1–H1A⋯O5: 2.613 Å, 3.339 Å, 132.69°) and van der Waals interactions (Fig. 2d).

### Crystal structural of [CuL(H_2_O)] (3)

**Fig. 3:**
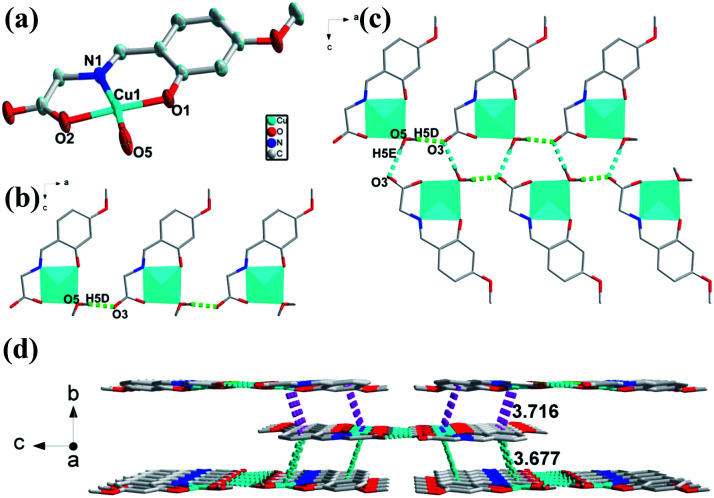
(a) Views of 3 ORTEP representation showing the local coordination environment around the Cu(ii) centre with 30% thermal ellipsoid probability. Hydrogen atoms have been omitted for clarity. (b) The 1D chain structure of 3 with H-bonding from *b* axis. (c) The 1D chain structure of 3 with H-bonding from b axis. (d) Viewing of the 3D structure with π–π stacking from *b* axis.

The crystal structure of 3 crystalizes in orthorhombic *Pbca* space group. Fig. 3a presents the asymmetric unit of 3, which is composed of one Cu(ii) ion, one deprotonated ligand and one coordinate water molecule. The four-coordinated Cu atom are surrounded by nitrogen and oxygen atoms with distorted quadrilateral coordination geometry, consisting of one nitrogen atom (Cu–N bond length of Cu(1)–N(1) is 1.922 Å) and two oxygen atoms (Cu–O bond length of Cu(1)–O(1), Cu(1)–O(2) are 1.883, 1.931 Å respectively) from one ligand and one oxygen atom from water molecule (Cu–O bond length of Cu(1)–O(5) is 1.933 Å). The two adjacent mononuclear structures formed by one Cu(ii) ion and one ligand are bridged by H-bonds (O5–H5D⋯O3: 1.838 Å, 2.672 Å, 170.09°) to result a 1D chain structure (Fig. 3b). The adjacent two chains are constructed by H-bonds (O5–H5E⋯O3: 1.956 Å, 2.803 Å, 178.31°) to form a double-stranded 1D supramolecular structure (Fig. 3c). Furthermore, the 3D structures are extended *via* π–π stacking interactions (3.677 Å and 3.716 Å) packing from 1D chains (Fig. 3d).

### Structural conversion of 1, 2 and 3

**Fig. 4:**
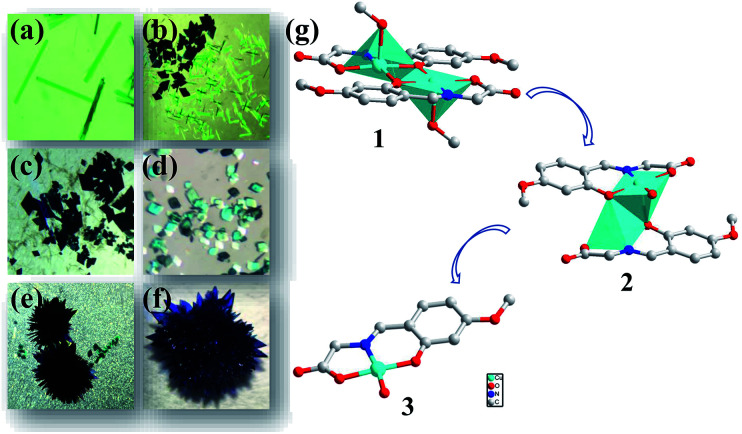
Time-series snapshot images for 1, 2 and 3 in the mother solution. (a) Rod-shaped crystals of 1 obtained after 2 days. (b and c) Rod-shaped crystals of 2 disappear gradually and the number and size of the rhombus-shaped crystals of 2 increase after 4 days. (d) Only the rhombus-shaped crystals of 2 exit after 5 days. (e) 2 disappear gradually and the number and size of the block of 3 increase. (f) Only the block crystals of 3 exit after 10 days. (g) The structural conversion between 1, 2 and 3.

It is noteworthy that the ligand and copper perchlorate formed three products with different crystal shapes and colours through coordination, 1 forming a rod-shaped green crystal, 2 forming a blue rhombic crystal and 3 forming a Prussian blue block crystal. The conversion between them occurred over time in the mother solution. It is known that two kinetic products and one thermodynamic product were formed step by step from the observation. When ligand and metal salts reacted in the mother solution, the three products could be identified by the naked eye due to their different morphology, and time-series snapshots of the crystal growth process could be recorded by optical photography (Fig. 4). In fact, as the mother solution slowly evaporated, the green rod-shaped crystal of 1 was obtained after two days, while the rod-shaped crystal remained in the mother solution and the blue rhomboid crystal of 2 appeared after three days. With the gradual reduction of the rod-shaped crystal, only the rhomboid crystal was finally completely formed. With the mother solution continued to evaporate slowly, the crystal was completely converted to Prussian blue block crystal after ten days. Single crystal X-ray crystallographic analysis reveals that 1, 2 and 3 show completely different structures. Although both 1 and 2 are binuclear molecular structures, the binuclear molecular structures are obviously different due to the different of the coordination solvent molecules and their positions. In the process of conversion from 1 to 2, the coordination solvent molecules were replaced by water molecules instead of methanol molecules. The reason is possible that the mother solution contains trace amount of water and the water molecule content takes the initiative in the evaporation process of the mother solution. With the further reduction of mother solution, Cu–O coordination bonds of 2 were fractured, resulting in the conversion of the binuclear to mononuclear structure, which shows that the mononuclear structure is the most stable in the series of structures. Going further, 2 and 3 respectively maintain the stable structure in water. When 2 was stored alone in the methanol solution, it was observed that the blue rhombic crystals were converted to green rod-shaped crystal within 10 min (Fig. S7†).

**Fig. 5:**
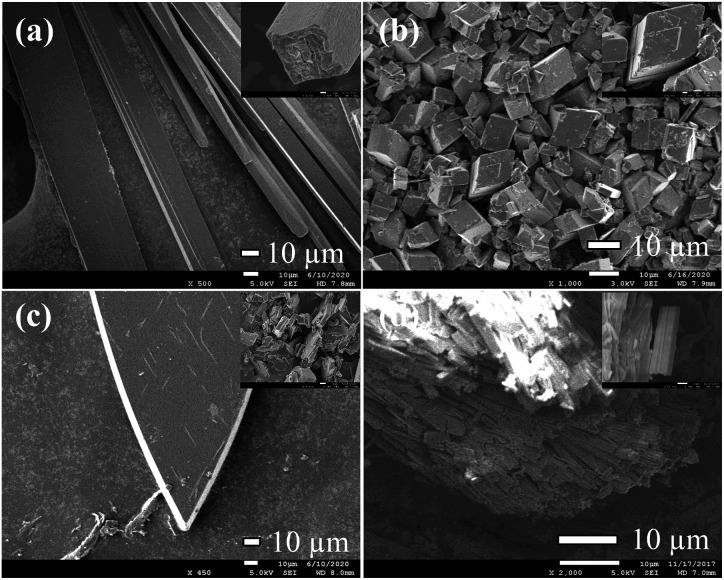
SEM of 1 (a), 2 (b), 3 (c) and 2 after soaking in MeOH solution (d).

Based on the studies of crystal structures, the morphologies of 1, 2, 3 were characterized with SEM in order to confirm the crystals conversion (Fig. 5). The resulting 1 showed a rod-like morphology consistent with that of snapshot image, which was related to the 1D chain arrangement (Fig. 5a). The SEM images of 2 revealed that the products are rhombic block structures with lamella stacked one by one (Fig. 5b). Moreover, petaloid crystal 3 converted by 2 showed gradual lamellar growth, resulting in the formation of dense and smooth block structures (Fig. 5c). Importantly, the reversible conversion of 1 and 2 can be observed by SEM combining with PXRD (Fig. S8†). After soaking 2 in methanol solution for 10 min, the rhombic morphology was obviously transformed into small rod-shaped stacking structures (Fig. 5d).

**Fig. 6:**
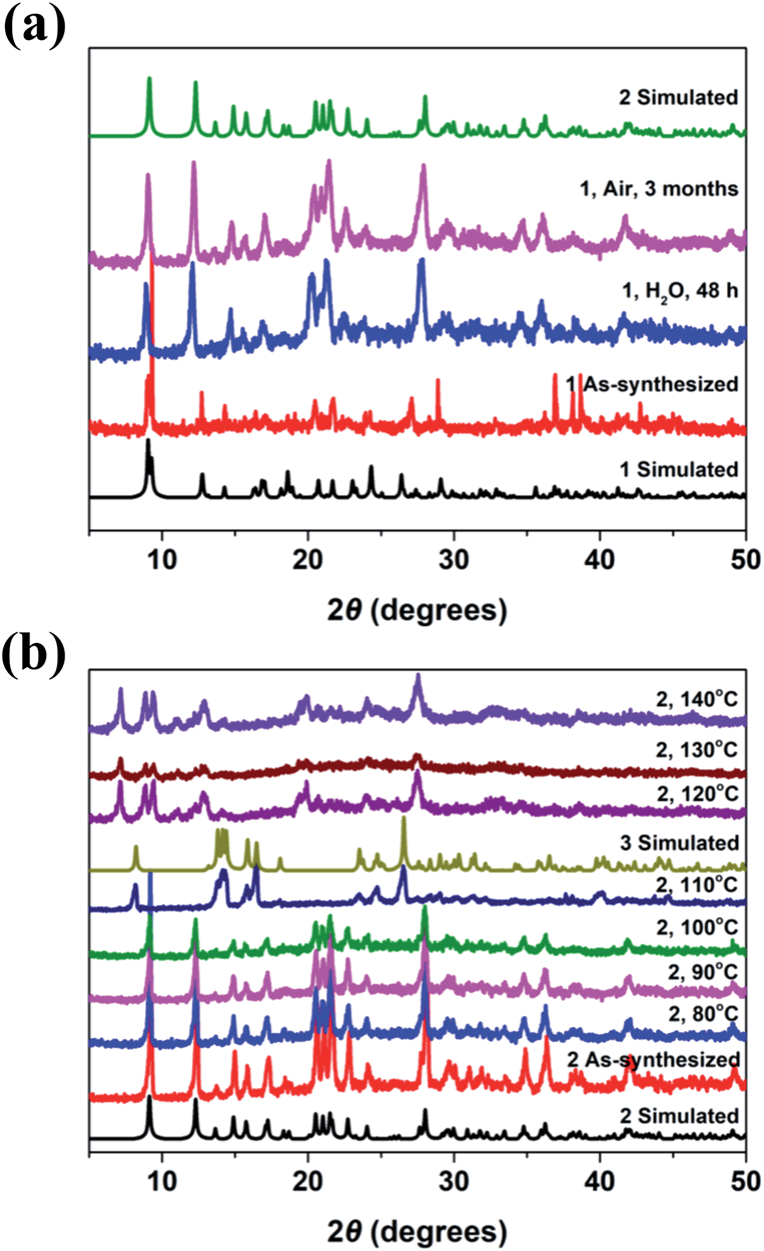
(a) Powder X-ray diffraction patterns of complex 1 and that immersed in water. (b) Temperature-dependent Powder X-ray diffraction patterns of complex 2 and that at different temperature.

In order to further demonstrate the crystal conversion of complexes 1–3, the time – dependent and temperature – dependent PXRD spectra was investigated. As shown in Fig. 6a, the PXRD peak of complex 1 changed after immersion in water for 48 hours, which was consistent with the PXRD fitting peak of complex 2, indicating conversion from 1 to 2. It can be seen that methanol molecules coordinate with metal ions in the axial direction based on the crystal structure, while the axial coordination bond is relatively long and easy to break, so the methanol is easy to lose after the invasion of water molecules, which lead to the conversion of crystal structure. On the other hand, the PXRD peak of complex 1 changed after exposure to air for three months, which was also consistent with the PXRD fitting peak of complex 2, indicating that the crystal of 1 could be transformed to 2 slowly under the influence of water molecules in air.

As shown in Fig. 6b, the pattern of PXRD peak of complex 2 was almost remained with the temperature rising to 100 °C. While, it has been changed obviously and was consistent with the fitting peak of complex 3 when the sample was heated to 110 °C, which indicated that the coordination bond fracture occurred resulting in structural transformation. It is occupied by the phenolic hydroxyl O atom of the ligand in the axial direction in the crystal structure of complex 2, which can convert to complex 3 because of the axial coordination bond is easier to break. It was found that the diffraction peak changed obviously when the sample was placed at 120–140 °C, which was due to the coordination water molecules lost combined with the TGA at this temperature and led to the transformation of the crystal structures.

## Conclusions

A new kind of tridentate ligand has been designed and synthesized. One of the advantages of these kinds of ligand are the open coordination sites of their metal coordination complexes, which provides the platform to produce the dynamical control of compositions and structures of coordination complexes. It is very important for us to understand the mechanism not only for coordination reaction toward the expectant complexes with functional properties, but also for the dynamical response to the external stimulations. In this work, we have successfully to observe the three different Cu(ii) complexes ([Cu_2_(L)_2_(MeOH)_2_] (1), [Cu_2_(L)_2_(H_2_O)_2_] (2) and [CuL(H_2_O)] (3)) in one beaker. Also, the reversible conversion of complexes 1 and 2 has been realized kinetically. The results will contribute to the controllable synthesis of the functional coordination complexes.

## Conflicts of interest

There are no conflicts to declare.

## Supplementary Material

RA-010-D0RA07231A-s001

RA-010-D0RA07231A-s002
