# Supplementary material for: Structural conversion of three copper(ii) complexes with snapshot observations based on the different crystal colours and morphology
Source: RSC Adv. 2020 Nov 26;10(70):42964–70. doi: 10.1039/d0ra07231a (PMC9058286; doi:10.1039/d0ra07231a)
Supplement: RA-010-D0RA07231A-s001 [file RA-010-D0RA07231A-s001.pdf]

Electronic Supplementary Material (ESI) for RSC Advances.
This journal is © The Royal Society of Chemistry 2020

Supporting Information

Structural conversion of three Copper(II) complexes with snapshot observation based on the different crystal colours and morphology

Hao Su[a], Zhongkui Li,[a] Junrui Tan,[a] Hongwei Ma,[b] Li Yan[b] and Hui Li*[a]

[a] *Key Laboratory of Cluster Science of Ministry of Education, School of Chemistry and Chemical Engineering, Beijing Institute of Technology, Beijing 100081, P. R. China*

*E-mail: lihui@bit.edu.cn

[b] *Analytical and Testing Center, Beijing Institute of Technology, Beijing 102488, China*

Table of Content

S1. Characterization of L, 1, **2** and **3.**

Fig. S1. ^{1}H NMR spectra of **L**.

Fig. S2. IR spectra of **L, 1**, **2** and **3**.

Fig. S3. Coordination modes of the metal in the compounds **1**(a), **2**(b) and **3**(c).

Fig. S4. Simulation and experiment PXRD pattern of **1**, **2** and **3**.

Fig. S5. TG curves of **1**, **2** and **3**.

S2. Crystal Structure Information List of 1, **2** and **3.**

Table S1. Selected bond distances (Å) and angles (°) for **1**.

Table S2. Selected bond distances (Å) and angles (°) for **2**.

Table S3. Selected bond distances (Å) and angles (°) for **3**.

S3. Crystal Structure and conversion of 2.

Fig. S6. 2D layer structure of **2**.

Fig. S7. The rhombus-shaped crystals of **2** convert quickly to rod-shaped crystals of 1 in methanol solution within 10 min.

Fig. S8. PXRD pattern of **2** after soaking in MeOH solution.

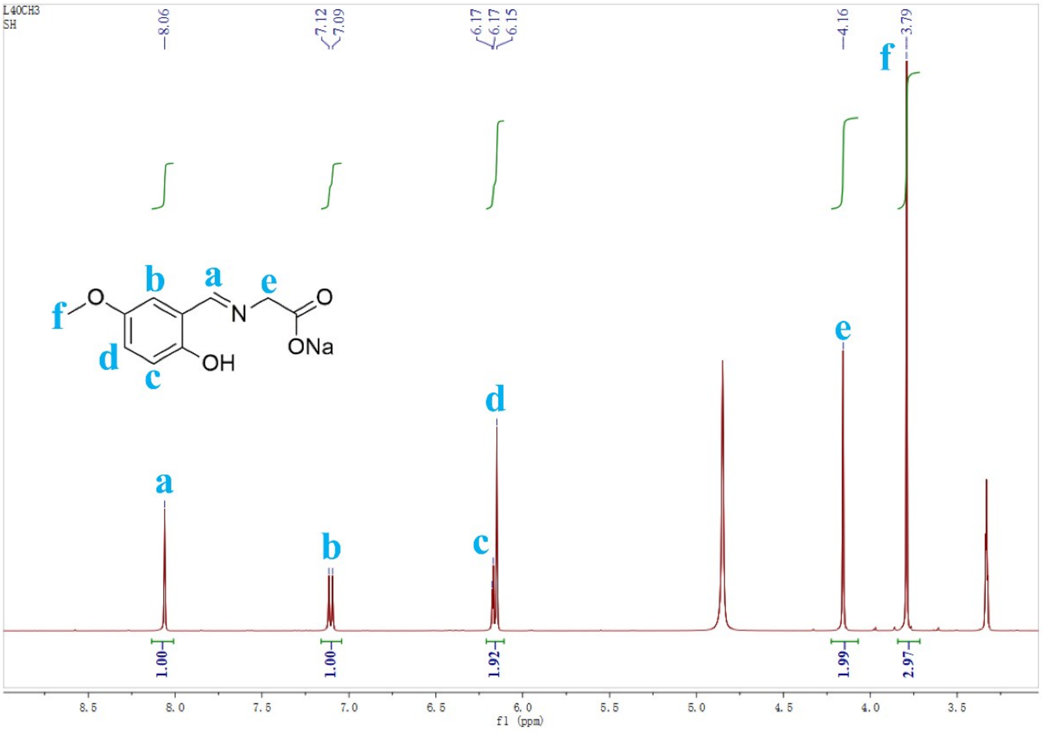

Fig. S1. ^{1}H NMR spectra of **L**.

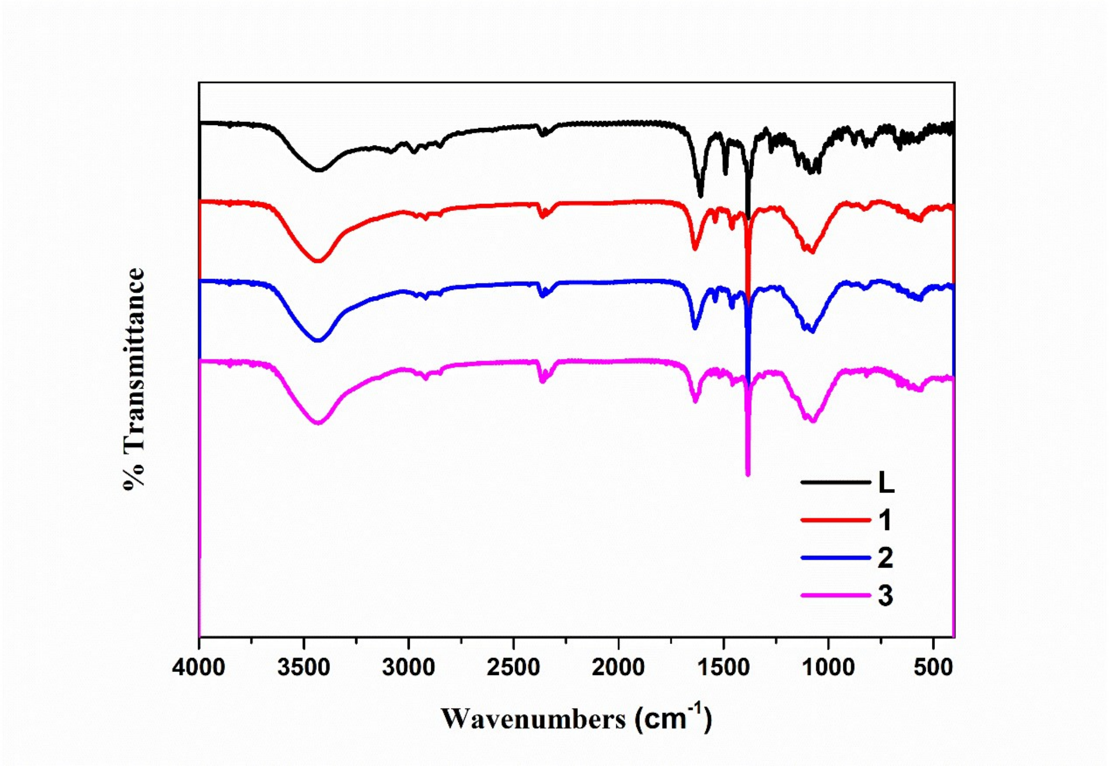

Fig. S2. IR spectra of **L, 1**, **2** and **3**.

(a) (b) (c)

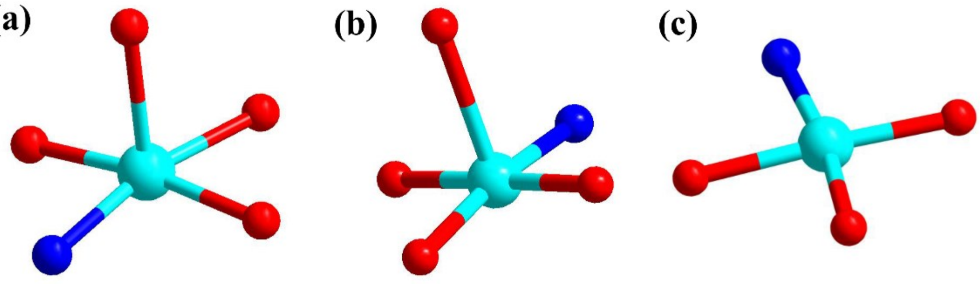

Fig. S3. Coordination modes of the metal in the compounds **1**(a), **2**(b) and **3**(c).

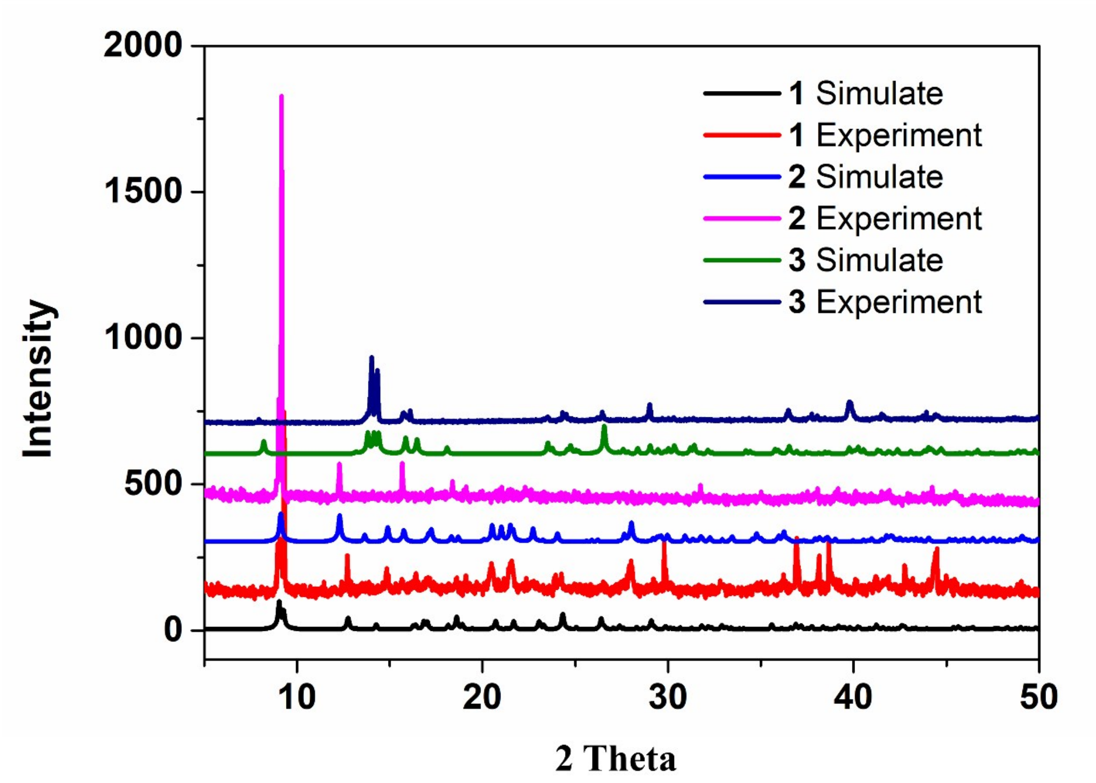

Fig. S4. Simulation and experiment PXRD pattern of **1**, **2** and **3**.

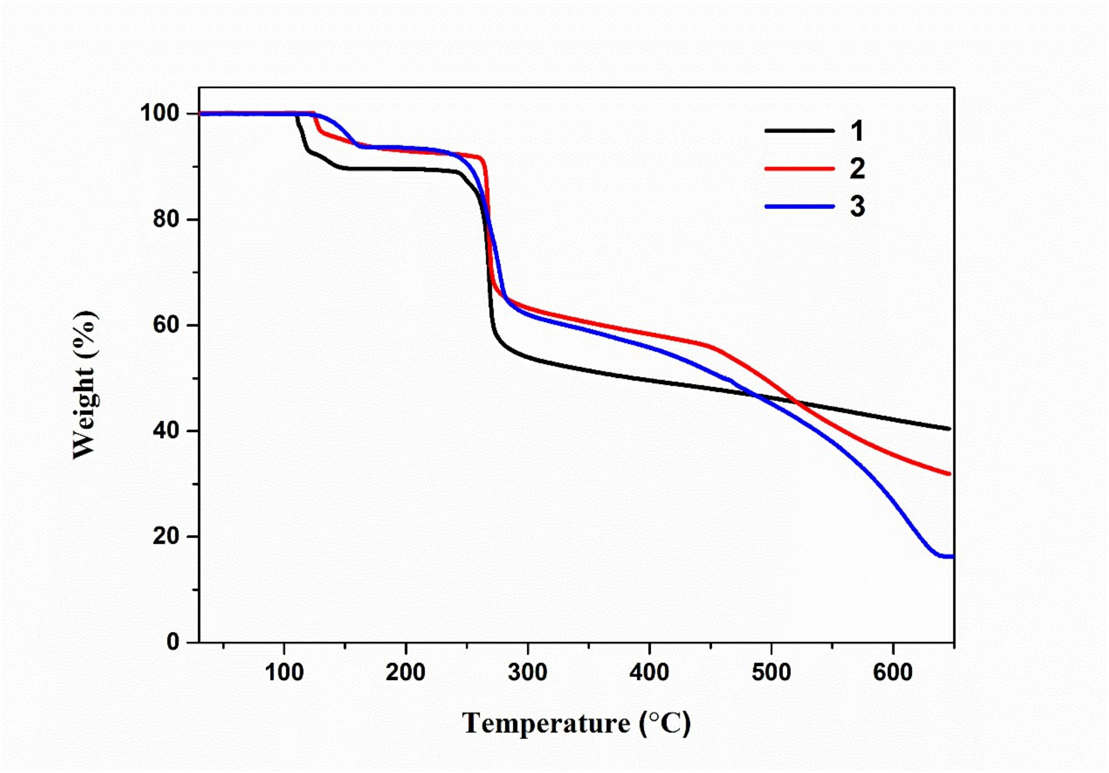

Fig. S5. TG curves of **1**, **2** and **3**.

Table S1. Selected bond distances (Å) and angles (°) for **1**.

Cu(1)–N(2)	1.929(2)	Cu(1)–O(2)	1.958(2)
Cu(1)–O(1)	1.9659(18)	Cu(1)–O(1)	1.9983(18)
Cu(1)–O(5)	2.252(2)		
N(2)–Cu(1)–O(2)	82.94(9)	N(2)–Cu(1)–O(1)	93.11(8)
O(2)–Cu(1)–O(1)	171.02(8)	N(2)–Cu(1)–O(1)	167.65(9)
O(2)–Cu(1)–O(1)	103.59(8)	O(1)–Cu(1)–O(1)	78.88(8)
N(2)–Cu(1)–O(5)	100.02(9)	O(2)–Cu(1)–O(5)	93.53(9)
O(1)–Cu(1)–O(5)	95.10(8)	O(1)–Cu(1)–O(5)	90.13(8)

Table S2. Selected bond distances (Å) and angles (°) for **2**.

Cu(1)–O(3)	1.906(6)	Cu(1)–N(1)	1.920(8)
Cu(1)–O(2)	1.959(6)	Cu(1)–O(1)	1.962(6)
Cu(1)–O(3)	2.433(6)		
O(3)–Cu(1)–N(1)	94.9(3)	O(3)–Cu(1)–O(2)	85.2(3)
N(1)–Cu(1)–O(2)	171.2(3)	O(3)–Cu(1)–O(1)	177.5(3)
N(1)–Cu(1)–O(1)	83.7(3)	O(2)–Cu(1)–O(1)	95.8(3)
O(3)–Cu(1)–O(3)	90.1(2)	N(1)–Cu(1)–O(3)	95.0(3)
O(2)–Cu(1)–O(3)	93.8(3)	O(1)–Cu(1)–O(3)	92.2(2)

Table S3. Selected bond distances (Å) and angles (°) for **3**.

Cu(1)–O(1)	1.883(2)	Cu(1)–N(1)	1.921(2)
Cu(1)–O(2)	1.931(2)	Cu(1)–O(5)	1.933(2)
O(1)–Cu(1)–N(1)	95.62(9)	O(1)–Cu(1)–O(2)	176.21(10)
N(1)–Cu(1)–O(2)	84.01(10)	O(1)–Cu(1)–O(5)	93.52(10)
N(1)–Cu(1)–O(5)	169.80(10)	O(2)–Cu(1)–O(5)	87.16(10)

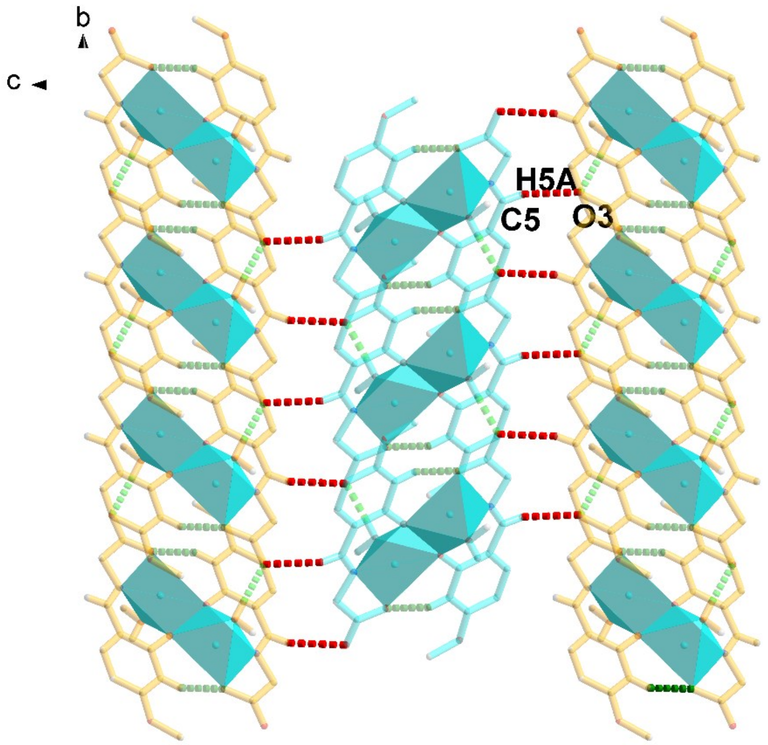

Fig. S6. 2D layer structure of **2**.

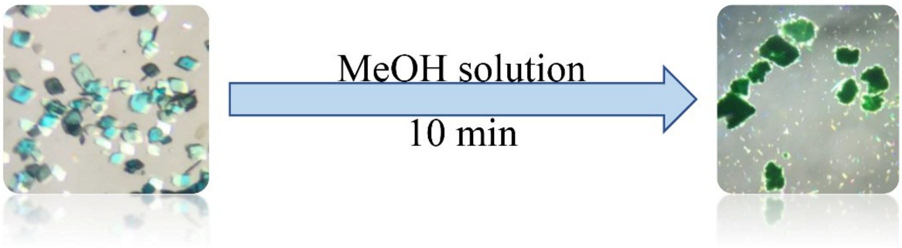

Fig. S7. The rhombus-shaped crystals of **2** convert quickly to rod-shaped crystals of **1** in methanol solution within 10 min.

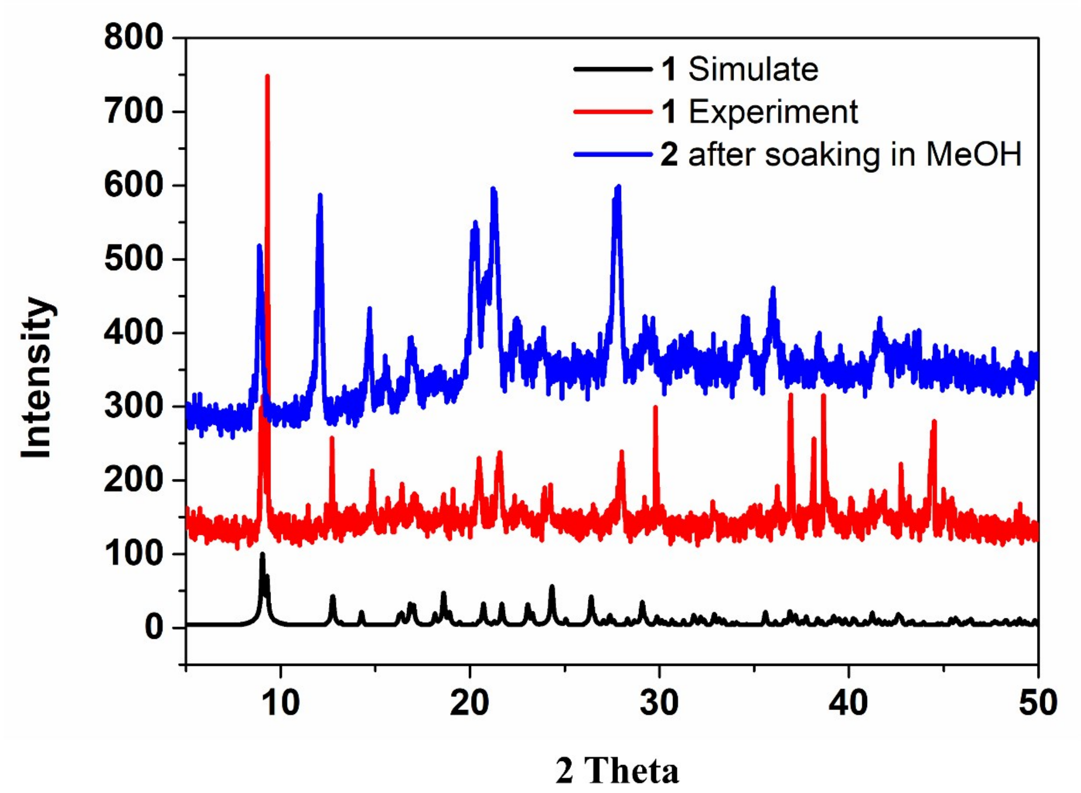

Fig. S8. PXRD pattern of **2** after soaking in MeOH solution.
